# Tissue‐specific differences in Ca^2+^ sensitivity of the mitochondrial permeability transition pore (PTP). Experiments in male rat liver and heart

**DOI:** 10.14814/phy2.16056

**Published:** 2024-05-22

**Authors:** Carolina Ricardez‐Garcia, Mauricio Reyes‐Becerril, Edson Mosqueda‐Martinez, Ofelia Mendez‐Romero, Angelica Ruiz‐Ramírez, Salvador Uribe‐Carvajal

**Affiliations:** ^1^ Departamento de Genética Molecular, Instituto de Fisiología Celular Universidad Nacional Autónoma de México, Ciudad Universitaria Mexico City Mexico; ^2^ Departamento de Biomedicina Cardiovascular Instituto Nacional de Cardiología Ignacio Chávez Mexico City Mexico

**Keywords:** calcium, heart mitochondrial, liver mitochondrial, mitochondrial permeability transition, reactive oxygen species, tissue specificity

## Abstract

Permeability transition pore (PTP) opening dissipates ion and electron gradients across the internal mitochondrial membrane (IMM), including excess Ca^2+^ in the mitochondrial matrix. After opening, immediate PTP closure must follow to prevent outer membrane disruption, loss of cytochrome *c*, and eventual apoptosis. Flickering, defined as the rapid alternative opening/closing of PTP, has been reported in heart, which undergoes frequent, large variations in Ca^2+^. In contrast, in tissues that undergo depolarization events less often, such as the liver, PTP would not need to be as dynamic and thus these tissues would not be as resistant to stress. To evaluate this idea, it was decided to follow the reversibility of the permeability transition (PT) in isolated murine mitochondria from two different tissues: the very dynamic heart, and the liver, which suffers depolarizations less frequently. It was observed that in heart mitochondria PT remained reversible for longer periods and at higher Ca^2+^ loads than in liver mitochondria. In all cases, Ca^2+^ uptake was inhibited by ruthenium red and PT was delayed by Cyclosporine A. Characterization of this phenomenon included measuring the rate of oxygen consumption, organelle swelling and Ca^2+^ uptake and retention. Results strongly suggest that there are tissue‐specific differences in PTP physiology, as it resists many more Ca^2+^ additions before opening in a highly active organ such as the heart than in an organ that seldom suffers Ca^2+^ loading, such as the liver.

## 1 INTRODUCTION

In response to different stimuli, large fluctuations in Ca^2+^ concentration are observed both in the cytosol [Ca^2+^]_cyt_ and in mitochondria [Ca^2+^]_Mit_ (Campbell, 2014; Carafoli, 1987; Romero‐Garcia & Prado‐Garcia, 2019) modulating many processes including oxidative phosphorylation (Bagur & Hajnóczky, 2017). While the basal [Ca^2+^]_cyt_ is slightly under 100 nM, upon stimulation it may rise more than 10 times, to 1 μM in liver (Gunter et al., 1998) or 1.7 μM in the heart (Lu et al., 2016). The mitochondrial Ca^2+^ uniporter (MCU) transports Ca^2+^ into the mitochondrial matrix (Kovacs‐Bogdan et al., 2014; Vais et al., 2020), while a slower Ca^2+^ return to the cytoplasm is mediated by antiport with either Na^+^ (mostly in heart) or H^+^ (mostly in liver) (Belosludtsev et al., 2019). In fact, there are Ca^2+^ hotspots in regions where the endoplasmic reticulum/mitochondrion come near, thus explaining the large amount of Ca^2+^ needed to elicit half‐activity by the Ca^2+^ uniporter. In the extracellular compartment Ca^2+^ is 10^4^ times more concentrated than in the cytoplasm and its uptake makes it an ideal second messenger (Campbell, 2014). In addition, in different tissues the rate of mitochondrial Ca^2+^ uptake is different, for example, Ca^2+^ uptake in heart is faster than in liver due to low expression of the Ca^2+^ uniporter control subunits MICU1 and MICU2 as compared to the channel subunit MUC resulting in a low MICU1/MUC ratio (Paillard et al., 2017). Thus, increasing MICU1 expression changes Ca^2+^ uptake kinetics in the heart to make it comparable to the liver (Paillard et al., 2017).

In mitochondria, Ca^2+^ uptake is faster than Ca^2+^ efflux, so it may reach high concentrations in the mitochondrial matrix during high cellular activity (Basso et al., 2018; Williams et al., 2013). This seems to be solved by opening the IMM permeability transition pore (PTP). PTP allows passage of molecules up to 1.5 kDa, collapsing the electric transmembrane potential (ΔΨ) and allowing [Ca^2+^]_Mit_ to diffuse to the cytoplasm (Balakirev & Zimmer, 1998; Haworth & Hunter, 1979; Hurst et al., 2017). The mitochondrial uptake of Ca^2+^ promotes PTP opening, which is partially inhibited by ADP (Halestrap et al., 1997; Haworth & Hunter, 1979, 2000; Zazueta et al., 2010). While PTP opening may be reversible, irreversible‐opening of PTP has been associated to the cell death due to outer mitochondrial membrane disruption and eventual release into the cytoplasm of proteins such as cytochrome *c* and the apoptosis‐inducing factor (AIF) (Bauer & Murphy, 2020; Crompton, 1999; Kroemer et al., 2007; Petronilli et al., 2001). In contrast, rapid PTP opening/closing, that is flickering seems to prevent cell damage (Kim et al., 2003; Rottenberg & Hoek, 2021). Thus, while PTP opening prevents ROS overproduction and Ca^2+^ overload, PTP closure prevents ATP depletion (Cadenas, 2018; Morales‐García et al., 2021).

For years the molecular identity of PTP has been investigated. Proteins like voltage‐dependent anion channel (VDAC), Cyclophilin D (CypD), the mitochondrial phosphate carrier (PiC), hexokinase, the adenine nucleotide translocator (ANT), and F_1_F_o_‐ATP synthase have been proposed to form PTP, although a firm conclusion still eludes researchers (Bernardi et al., 2021; Gutiérrez‐Aguilar & Baines, 2015; Izzo et al., 2016; Mnatsakanyan et al., 2017; Strubbe‐Rivera et al., 2021). Recently, in regard to PTP structure it was suggested that there may at least two PTPs, one constituted by ANT and another by ATP synthase (Carrer et al., 2021; Neginskaya et al., 2022, 2023).

From the above, it may be proposed that together with the detoxifying role of PTP opening, its efficient closure is needed for survival. Indeed, flickering has been reported in heart and muscle mitochondria (Boyman et al., 2019; Korge et al., 2011; Lu et al., 2016). Still, it is not known if PTP dynamics differ from tissue to tissue, that is whether in tissues that seldom see variations in [Ca^2+^]_Mit_, such as the liver (Bagur & Hajnóczky, 2017; Burgess et al., 1984; Carafoli, 1987), PTP is as resilient as in tissues that are subjected to large, frequent Ca^2+^ fluxes such as the heart (Abelmann, 1961; Akopova, 2001; Chalmers & Nicholls, 2003; Zhong et al., 2008). Here, PTP reversibility in isolated murine mitochondria from either heart or liver was compared to determine whether PTP properties vary from tissue to tissue. Heart mitochondria (Heart_Mit_) PT remained reversible for longer periods and under higher Ca^2+^ concentrations than liver mitochondria (Liver_Mit_). Our results may have profound implications concerning the physiological role of the mitochondrial PTP in different organs.

## 2 MATERIALS AND METHODS

### 2.1 Reagents

All the chemicals used were analytic grade. Sucrose (S0389), CaCl_2_ (C5080), succinic acid (S7501), L‐glutamic acid (G12519), malic acid (M0625), EGTA (E0396), ADP (01905), safranine‐O (S2255), and Arsenazo III (A92775) were from Sigma Chem Co (MO, USA). Bovine serum albumin was from GoldBio (E217100, MO, USA). H_3_PO_4_, KCl and MgCl_2_ were from J.T. Baker Chemical Co. (NJ., USA), Ruthenium red was from Merck KGaA (R2751, Darmstadt, Germany). Cyclosporin A was from Sandimmum (Sandoz, Basilea, Swiss). Calcium Green‐5N was from Thermo Fisher Scientific (C3737 MA, USA).

### 2.2 Animals

Wistar male rats weighing 200 g were fed with Laboratory Rodent Diet 5001 (LabDiet, Minnesota, USA), were obtained from our animal facilities at either the Instituto de Fisiología Celular (IFC, UNAM) or the Instituto Nacional de Cardiología (INC) and were housed at 22 ± 2°C on a 12 h/12 h light/dark cycle, with free access to a standard laboratory rodent chow and water. Before the experiment, each rat was fasted for 12 h and euthanized by decapitation. Experimental procedures using animals at the Instituto Nacional de Cardiología were authorized by the “Committee for the Care and Use of Laboratory Animals (C.I.C.U.A.L.)” with protocol number INC/CICUAL/008/2020. At IFC, UNAM, a local committee approved all experimental procedures according to Ethics in Animal Experimentation (SUV124‐19).

### 2.3 Isolation of mitochondria

Liver and heart were extracted from the same individual and placed in cold isolation buffer (250 mM sucrose, 10 mM Tris–HCl and 1 mM EDTA, pH 7.3). Both tissues were processed at the same time, but with different protocols. The differences were to the need to properly homogenize the highly resistant cardiac muscle (see below). In both tissues adequate respiratory controls were obtained, ensuring that membrane integrity was preserved (Corcelli et al., 2010; Pallotti & Lenaz, 2007). The heart was minced and incubated for 10 min in isolation buffer plus 1 mg proteinase K/mL, then it was centrifuged at 12063× *g* for 10 min, the pellet was washed with the same buffer before being homogenized with a Tissuemizer (model TR‐10, Tekmar, Breisgau, Germany) at 3000 rpm × 10 s three times alternating with 1 min rest on ice. The homogenate was centrifuged a 1477× *g* for 5 min and the supernatant was centrifuged at 12063× *g* for 10 min. The mitochondrial pellet was resuspended and incubated with BSA 0.2% × 10 min and finally centrifuged 12063× *g* for 10 min and resuspended in reaction buffer (250 mM sucrose, 10 mM HEPES, pH 7.4) (Correa et al., 2007). At the same time, the liver was minced in isolation buffer as the heart, homogenized using a Potter‐Elvehjem teflon homogenizer and centrifuged 1477× *g* for 5 min. Then the supernatant was centrifuged at 12063× *g* for 10 min. The pellet was suspended in reaction buffer (250 mM sucrose, 10 mM Tris–HCl and 20 μM EDTA, pH 7.4) (Gutiérrez‐Aguilar & Baines, 2015). Mitochondrial suspensions from each source were kept in an ice‐bath and used within 3 h. To ensure mitochondrial quality, respiratory controls were measured in an oximeter (see below) and mitochondria were used only when RC was at least 4.0 for liver or 6.0 for heart.

### 2.4 Mitochondrial swelling

Swelling was followed as the decrease in absorbance at 540 nm (Jung et al., 1997). We used a DW2000 Olis/Aminco spectrophotometer (GA, USA) in split mode. The concentrations of Ca^2+^ and EGTA used are indicated under each figure.

### 2.5 Oxygen consumption

Experiments were conducted using a high‐resolution oxygraph (Oroboros, Innsbruck, Austria) at 30°C with continuous magnetic stirring. Reaction buffer for Liver_Mit_ was 250 mM sucrose, 10 mM Tris–HCl and 20 μM EDTA, pH 7.4 (Gutiérrez‐Aguilar & Baines, 2015) and for Heart_Mit_ was 120 mM KCl and 10 mM HEPES pH 7.4 (Correa et al., 2007). In both experiments mitochondria (1 mg prot./mL) were added to a chamber containing 10 mM/10 mM glutamate/malate (G/M) and 5 mM phosphate (Pi). After measuring the basal O_2_ consumption, one pulse of Ca^2+^ was added and EGTA was added as indicated.

### 2.6 Determination of mitochondrial Ca^2+^ retention capacity using either Arsenazo III or Ca^2+^ Green‐5N

In order to measure Ca^2+^ fluxes in mitochondria, it was decided to use two alternative methods: the DW2C absorbance‐based indicator arsenazo III or the fluorescent indicator Ca^2+^ Green‐5 N. For each method we used different reaction mixtures in an effort to eliminate any possible artifacts from our experiments. (a) Arsenazo III. Measurements of mitochondrial Ca^2+^ retention capacity in mitochondrial suspensions (1 mg prot./mL) were performed at room temperature with continuous magnetic stirring in a DW2000 Olis/Aminco spectrophotometer in dual mode 675–685 nm (Petronilli et al., 1993; Scarpa & Azzi, 1968; Uribe & Devlin, 1994). The reaction mixture (150 mM sucrose, 50 mM KCl, 5 mM Tris, and 250 μM KH_2_PO_4_ pH 7.4) was supplemented with 50 μM Arsenazo‐III, 100 μM ADP, and NADH‐linked respiratory substrates (10 mM malate, 10 mM glutamate). Ca^2+^ was added in pulses 5 or 10 μM each 1.5 min (Petronilli et al., 1993; Scarpa & Azzi, 1968). (b) Calcium Green‐5 N. Either Heart_Mit_ or Liver_Mit_ (1 mg/mL) were incubated in the same reaction buffer plus glutamate/malate (5 mM/5 mM) and 100 nM Calcium Green‐5N (Thermos Fisher Scientific, Walthman, MA, USA) for 1 min (Correa et al., 2007). Subsequently, CaCl_2_ was added repeatedly as indicated until PT induction (Figure S3). Where indicated, either 10 μM CsA a mMPT inhibitor or 75 μM ruthenium red (RuR) a Ca^2+^ uniport inhibitor were added. Fluorescence was measured in a PerkinElmer LS50B spectrofluorometer (MA, USA) using a magnetically stirred quartz cuvette. Temperature was 30°C. And the end of each experiment, saturated concentrations of Ca^2+^ (3 M) and EGTA (400 μM) were used to titrate the sample to determine the maximun (*F*_max_) and minimal (*F*_min_) fluorescence respectively (not shown). Mitochondrial [Ca^2+^] was calculated as described by Amigo et al. (2017):

$$\left[Ca^{2+}\right]=\mathrm{Kd}\times \frac{F-F_{\min}}{F_{\max}-F}$$

where *F* is experimental fluorescence. As suggested by the manufacturer, the dissociation constant used was Kd = 14 μM.

### 2.7 Reactive oxygen species measurements

Reactive oxygen species were measured in using an Amplex Red Kit (Invitrogen, Molecular Probes, Carlsbad, CA, USA). Briefly, samples were: (1) Ca^2+^ loaded, adding 30 μM Ca^2+^ to Liver_Mit_ or 150 μM Ca^2+^ to Heart_Mit_ and incubating for 4 min; (2) CsA samples, adding 10 μM CsA plus the same Ca^2+^ concentrations as in 1; and (3) adding the same Ca^2+^ concentrations as in 1 and then, EGTA 30 μM for Liver_Mit_ and 150 μM for Heart_Mit_ at different times (30, 60, 90, 120, 180, and 240 s). In a 96‐well microplate, a 50 μg aliquot from each sample was placed into a well containing 20 μL working solution 10 μM Amplex red, 0.2 U/mL horseradish peroxidase and 0.2 U superoxide dismutase/mL in 250 mM sodium phosphate pH 7. (4) Final volume 100 μL. Fluorescence was measured at 571 and 585 nm in a POLARstar Omega detector (BGM LABTECH, Offenburg, Germany). Results were interpolated against a calibration curve (0–200 nM H_2_O_2_) to convert relative fluorescence units (RFU) to nm H_2_O_2_ (Mishin et al., 2020).

### 2.8 Statistical analysis

One‐Way Anova tests were performed. Data are presented as mean ± SD. Sample sizes are indicated in figure captions. Tests were considered significant at the 95% level of confidence (*p* < 0.05). GraphPad Prism version 8 (GraphPad Software, CA, USA) was used for statistical analysis and to draw graphs.

## 3 RESULTS

The heart undergoes frequent depolarization events, while in contrast, other tissues such as the liver seldom see any activity. It was thus hypothesized that mitochondria in each of these tissues should exhibit different sensitivity to the wide Ca^2+^ fluxes associated to cell depolarization. To test this, murine liver or heart mitochondria were isolated from the same individual. In each sample, mitochondrial PT and its reversibility were evaluated by measuring different activities.

### 3.1 Oxygen consumption

**FIGURE 1:**
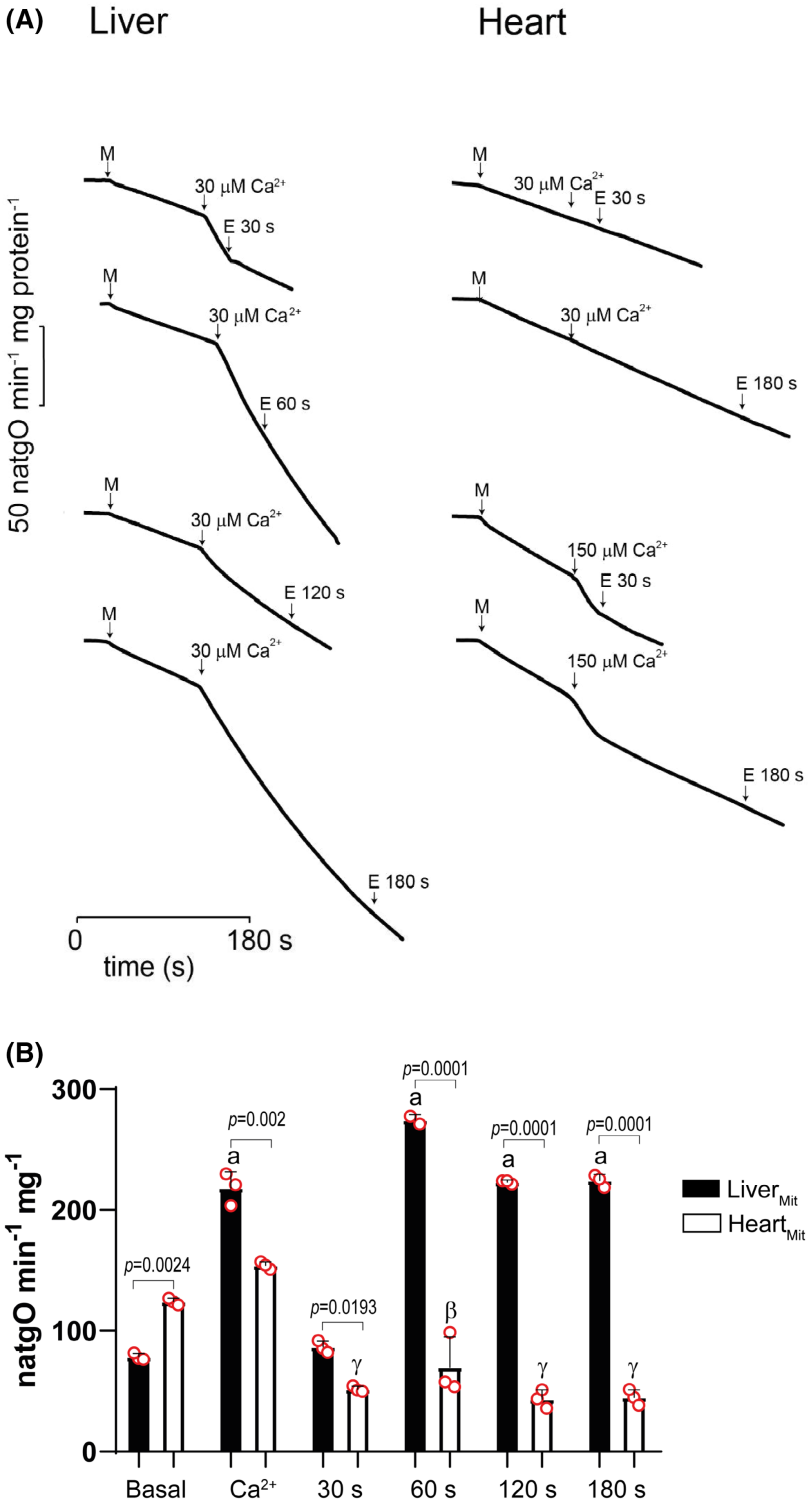
(A) Effect of Ca^2+^ addition and quenching on OCR measured as nanoatom‐gram of oxygen per minute per milligram of protein, (natgO min^‐1^ mg protein^‐1^). Liver_Mit_ (left) or Heart_Mit_ (right). Reaction mixture: 250 mM sucrose, 10 mM Tris, 5 mM Pi, 20 M EDTA, pH 7.4. Ca^2+^ addition was 30 M to Liver_Mit_ and either 30 or 150 M Ca^2+^ to Heart_Mit_ as indicated. EGTA was the same concentration as Ca^2+^. (B) Replot of data from triplicates of experiments in A. Liver_Mit_ (black columns) and Heart_Mit_ (white columns). Data are presented as mean ± SD of three independent experiments. Statistical differences from tissue to tissue are indicated in the figure with their *p* value. In addition, statistical differences within a tissue against its respective basal OCR are indicated as follows: Liver: latin letters; Heart, greek letters. For Liver_Mit_ “a”: *p* < 0.0001. For Heart_Mit_ “*α*”: *p* = 0.0243; “*β*”: *p* = 0.0003, and “*γ*”: *p* < 0.0001.

In isolated mitochondria, Ca^2+^ addition increases the O_2_ consumption rate (OCR). When Ca^2+^ is sequestered, the rate of O_2_ consumption returns to its basal levels unless PTP is open and depletes ΔΨ (Figure 1A) (Batandier et al., 2004; Zhong et al., 2008). In Liver_Mit_, 30 μM Ca^2+^ increased OCR and EGTA addition at 30 s reverted this increase. However, at 60 s or later EGTA was ineffective (Figure 1A Liver; Figure 1B, black columns). These results suggest that Liver_Mit_ PT became irreversible at some point after 30 s. When the same experiment was conducted in Heart_Mit_, 30 μM Ca^2+^ was also added and no effects on OCR were observed, EGTA had no further effects (Figure 1A Heart, two upper traces). Increasing added Ca^2+^ to 150 μM did accelerate OCR, which returned to the basal rate independently of EGTA addition reverted suggesting that PTP was closed (Figure 1A heart, two lower traces; Figure 1B white columns). Thus, in Liver_Mit_ PT was triggered by 30 μM Ca^2+^ and became irreversible sometime between 30 and 60 s, while in the Heart_Mit_ acceleration of O_2_ consumption was observed only at 150 μM Ca^2+^ and the rate of O_2_ consumption returned spontaneously to basal levels even after 180 s. Furthermore EGTA had no further effects. The results suggest that after 30 s of Ca^2+^ exposure, PTP became open in Liver_Mit_ while it remained closed in Heart_Mit_ (Figure 1).

### 3.2 Ca^2+^‐driven mitochondrial swelling

**FIGURE 2:**
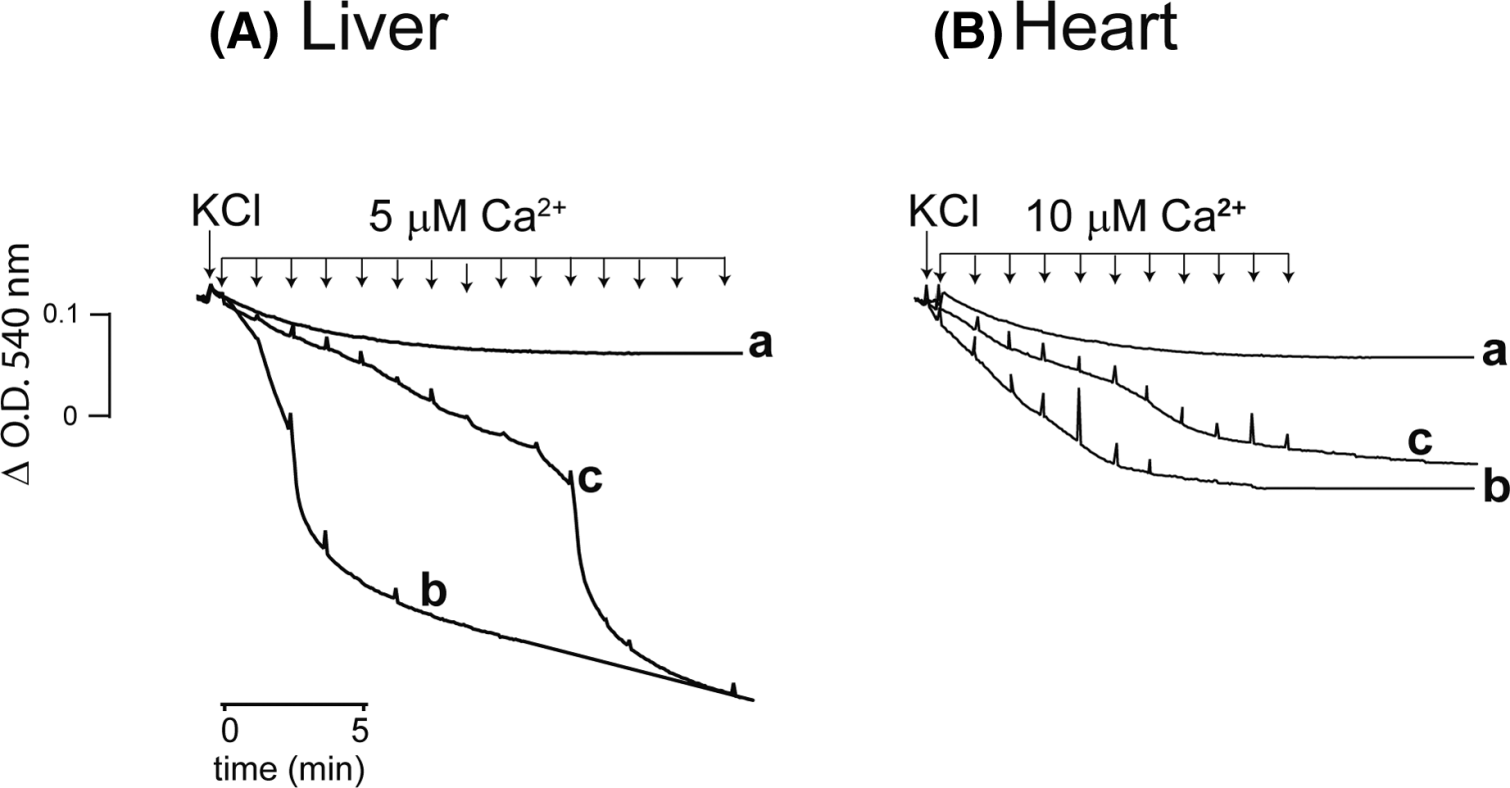
Mitochondrial swelling. Liver_Mit_ (A) is more sensitive than Heart_Mit_ (B). Reaction mixture as in Figure 1 plus 20 mM KCl. Swelling was induced with Ca^2+^ additions as indicated. (a) Control; no Ca^2+^ additions; (b) addition of Ca^2+^ pulses every 1.5 min (5 μM in liver and 10 μM Ca^2+^ in heart); (c) 10 μM CsA. Representative traces, *n* = 3 (for raw data see Figure S1).

**FIGURE 3:**
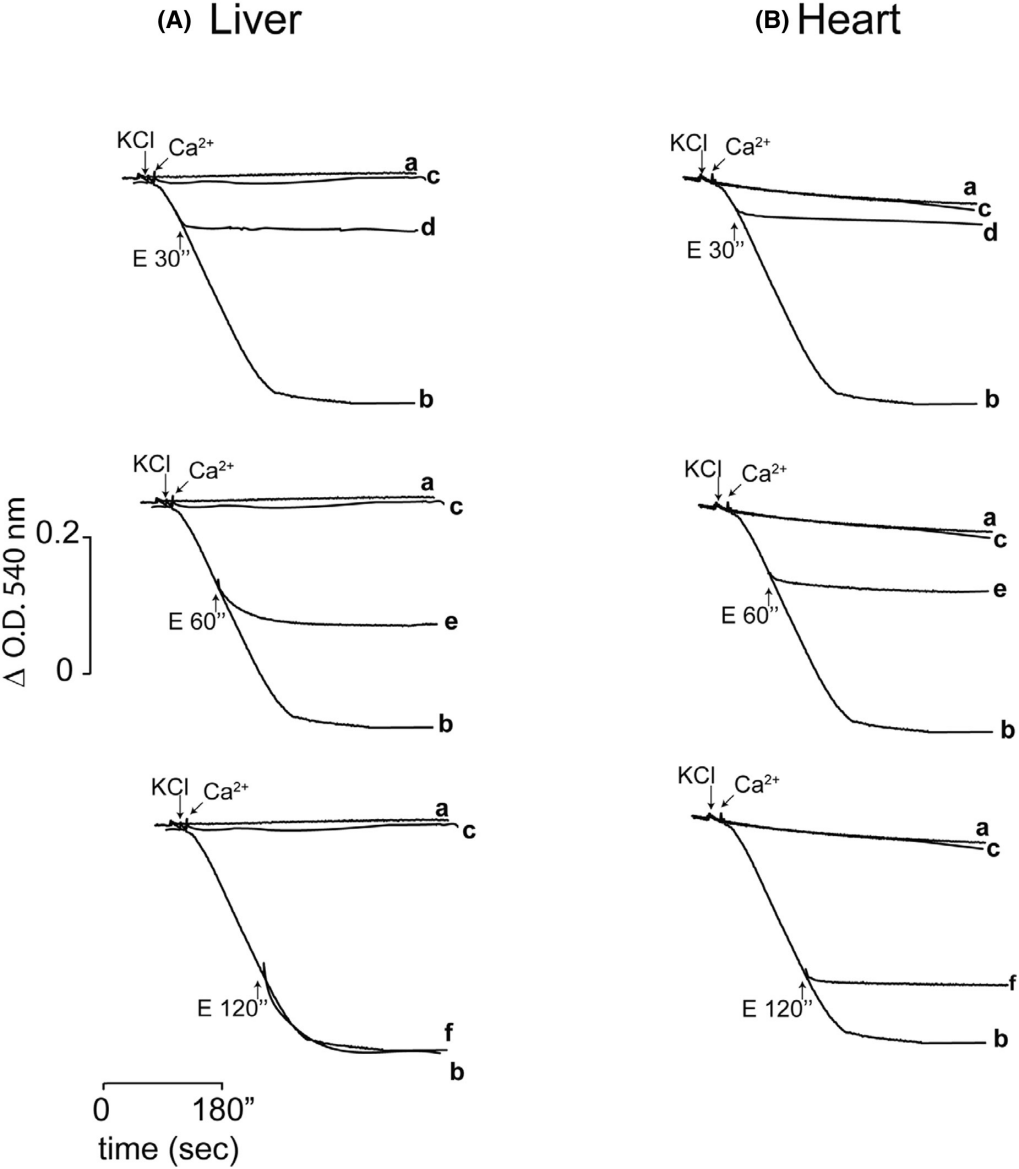
Mitochondrial (M) PT reversibility in (A) Liver_Mit_ and (B) Heart_Mit_. Reaction mixture: as in Figure 1. Swelling was induced with 20 mM KCl and swelling control was evaluated (a). PTP opening was induced with 30 μM Ca^2+^ in liver while in heart mitochondria 150 μM Ca^2+^ were needed (b). Where indicated 10 μM CsA (c). EGTA: 30 μM for Liver_Mit_ or 150 μM to heart_Mit_ at 30 (d), 60 (e) and 120 (f) sec (upward arrows). Representative traces, *n* = 3 (for raw data see Figure S2).

Ca^2+^ is taken avidly by mitochondria (Carafoli, 1987; Halestrap & Davidson, 1990) triggering PT which in the presence of high KCl, PT evokes swelling (Jung et al., 1997). Thus, to evaluate PT, Ca^2+^ was added to mitochondria in the presence of 20 mM KCl. Liver_Mit_ did not swell spontaneously (Figure 2A, trace a). However, sequential 5 μM Ca^2+^ additions led to rapid swelling beginning at the third addition (Figure 2A, trace b). In the presence of Cyclosporine A (CsA) swelling was inhibited partially (Figure 2A, trace c) further suggesting that swelling was due to PT. In control Heart_Mit_ swelling was minimal (Figure 2B, trace a). Under these conditions repeated addition of 10 μM Ca^2+^ induced mitochondrial swelling (Figure 2B, trace b). Again, CsA inhibited Heart_Mit_ swelling (Figure 2B, trace c). Thus, the results in Figure 2 confirm that mitochondrial swelling was more evident and took place at lower Ca^2+^ concentrations in liver than in Heart_Mit_. In addition when comparing to Figure 3B it was obvious that cardiac mitochondria did not undergo full swelling.

### 3.3 Reversibility of Ca^2+^‐driven mitochondrial PTP opening

In the presence of KCl, mitochondria exhibit only a mild rate of swelling (Figure 3, trace a); rapid mitochondrial swelling may be promoted by adding an adequate amount of Ca^2+^ (Figure 3, trace b); CsA prevents the Ca^2+^ effect, indicating that swelling is due to opening of PTP (Figure 3, trace c). In all cases, Ca^2+^ quenching should stop swelling by closing PTP. This system was tested in Liver_Mit_ and Heart_Mit_ to explore PT reversibility (Figure 3).

In Liver_Mit_ full swelling was observed at 30 μM Ca^2+^ while Heart_Mit_ needed 150 μM Ca^2+^ (Figure 3). In Liver_Mit_ Cyclosporine‐sensitive Ca^2+^‐mediated mitochondrial swelling was triggered with 30 μM Ca^2+^ (Figure 3A, trace b). Then, EGTA was added at different times, and it was observed that swelling stopped at 30 s (Figure 3A, trace d). When EGTA was added at 60 s (Figure 3A, trace e) swelling continued for 5 s and then stopped. At 120 s (Figure 3A, trace f) swelling was not inhibited. When the same experiment was conducted in Heart_Mit_, 150 μM Ca^2+^ was needed to trigger similar swelling (Figure 3B, trace b). In addition, at all times evaluated, 30, 60 or 120 sec, EGTA stopped swelling immediately (Figure 3B, traces d, e and f, respectively). Results suggest that while hepatic mitochondrial PTP opening lost reversibility after 30 s, cardiac mitochondrial PTP remained reversible after addition at all three times tested.

### 3.4 Ca^2+^‐retention assays using Arsenazo III

**FIGURE 4:**
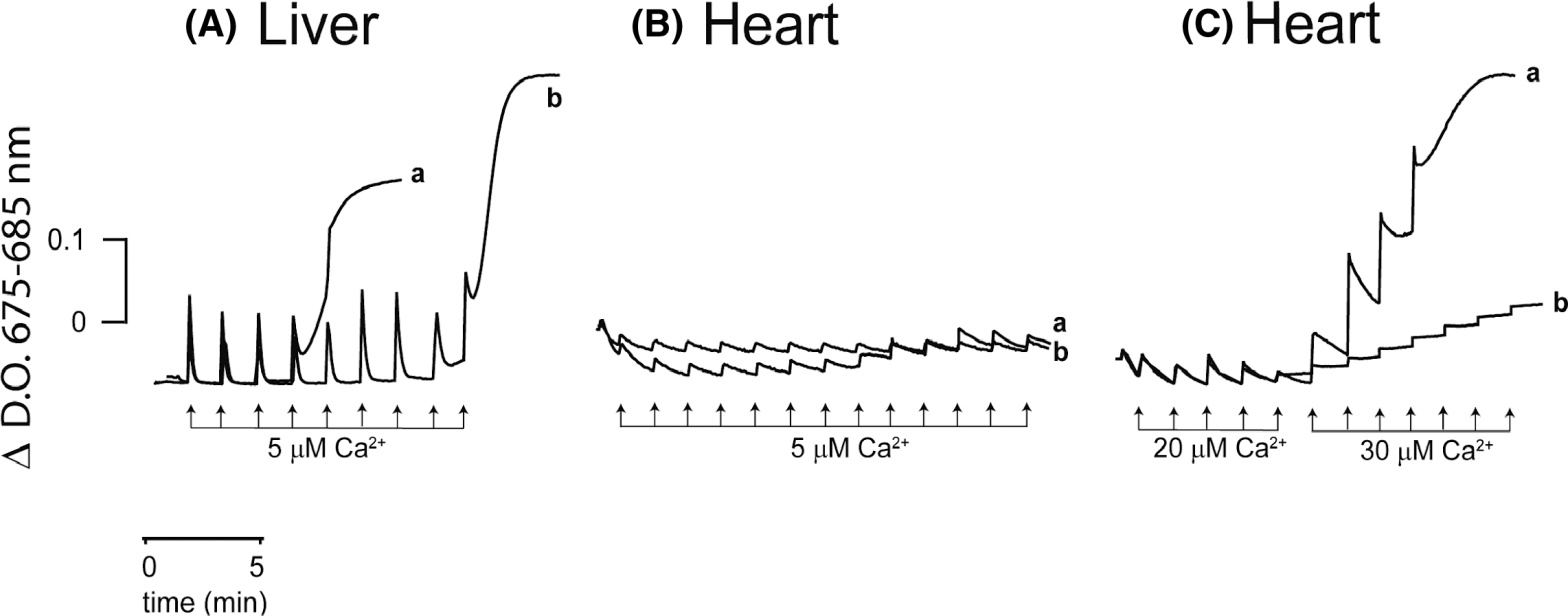
Calcium retention assays in isolated mitochondria (M). Effect of Cyclosporine A. (A) Liver_Mit_, (B) and (C) Heart_Mit_. Reaction mixture: as in Figure 1 except 50 μM Arsenazo III. Opening of PTP was induced adding 5 μM Ca^2+^ pulses in Liver_Mit_ or, as indicated, 5, 20 and 30 μM Ca^2+^ to Heart_Mit_ every 1.5 min (a). In traces b, 10 μM CsA. Representative traces, *n* = 3.

Many reports indicate that sequential Ca^2+^ additions trigger mitochondrial PT (Haworth & Hunter, 1979; Scarpa et al., 1978; Uribe & Devlin, 1994). As expected from Figures 2 and 3, in Liver_Mit_ the fourth to fifth 5 μM Ca^2+^ addition triggered PT and the release for Ca^2+^ (Figure 4A, trace a), while CsA delayed PT to about twice as many additions (Figure 4A, trace b). In contrast, in Heart_Mit_, repeatedly adding 5 μM Ca^2+^ with or without CsA did not trigger PT (Figure 4B). Only when adding aliquots of 20 μM and 30 μM Ca^2+^ did PTP open at 100 μM Ca^2+^ (Figure 4C, trace a). In addition, CsA delayed PT, even when 300 μM Ca^2+^ was added (Figure 4C, trace b). Again, our results indicate that PTP in Heart_Mit_ withstands much higher Ca^2+^ loading than Liver_Mit_ and it remains reversible for much longer.

### 3.5 Ca^2+^‐retention assays using Ca^2+^‐Green

In order to confirm our data with Arsenazo III, Ca^2+^ flows were measured with the fluorescent dye Ca^2+^ Green‐5N. In addition, a different reaction mixture with high KCl, and without previous treatment with chelex‐100 was used (Correa et al., 2007) (Figure S3). Sequential additions of 20 μM Ca^2+^ were tested: Liver_Mit_ presented PT at the fifth addition (Figure S3A, black trace a) while Heart_Mit_ resisted almost three times as many Ca^2+^ additions before undergoing PT (Figure S1A, gray trace b). When a higher Ca^2+^ (50 μM additions) was tested (Figure S3B), PT was exhibited at the second addition by Liver_Mit_ (Figure S3B, black trace a) while Heart_Mit_ underwent PT only the sixth to seventh addition (Figure S3B, gray trace b). Thus, the results were similar to those performed with Arsenazo III (Figure 4). Additionally, it was also confirmed that CsA inhibited PT in both cases (Figure S3C); except that in Figure S3C, an upward shift of Ca^2+^ was observed in Heart_Mit_ (Figure S3C, gray trace b) which was not present in the Arsenazo III experiment (Figure 4B). This was probably due to the addition of higher Ca^2+^ used in the Ca^2+^‐Green experiment and an active Ca^2+^ antiport in the heart. Indeed, RuR inhibited Ca^2+^ uptake (Figure S3D) by both Liver_Mit_ (Figure S3D, black trace a) and Heart_Mit_ (Figure S3D, gray trace b).

**FIGURE 5:**
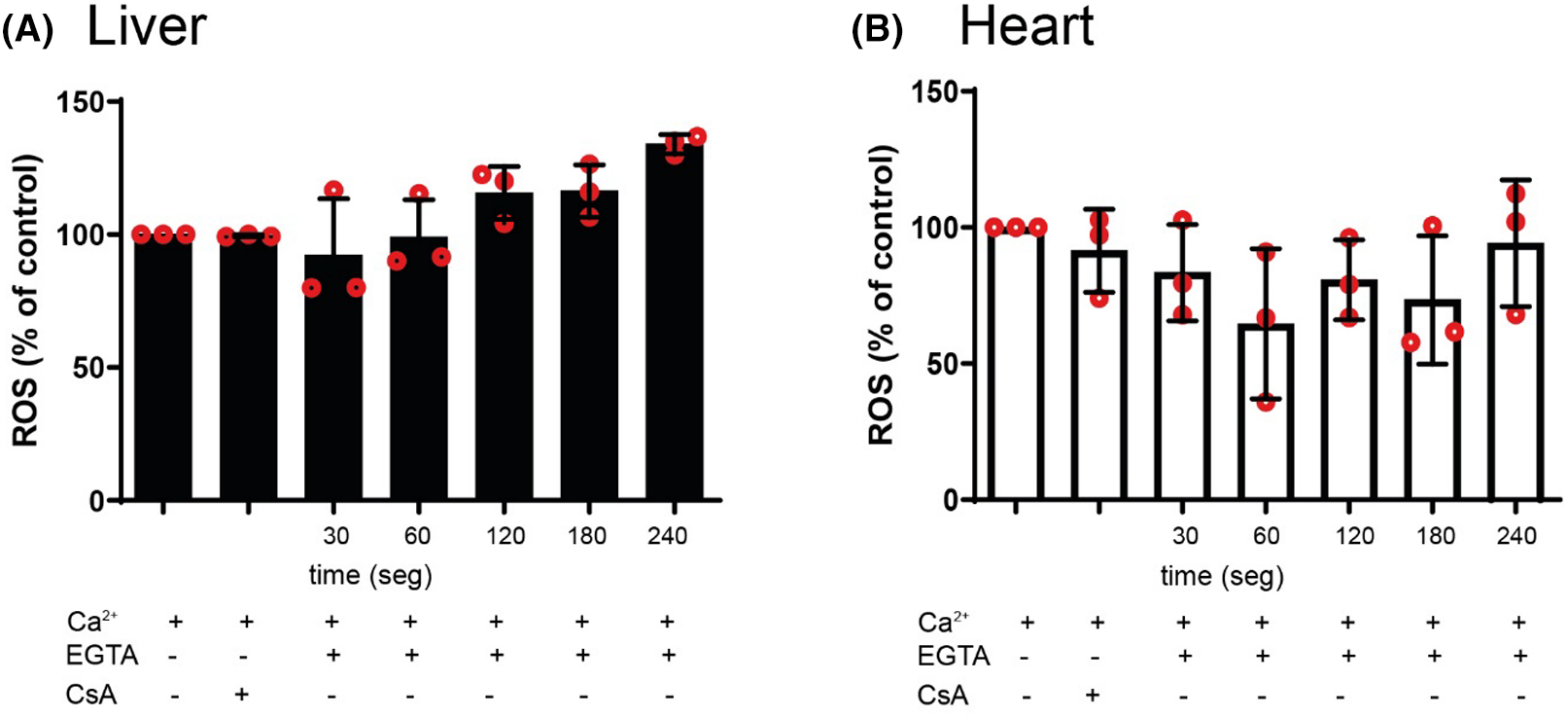
PT decreases ROS production in (B) Heart_Mit_ but not in (A) Liver_Mit_. Reaction mixture: As in Figure 1. PTP opening o was induced with 30 μM Ca^2+^ in Liver_Mit_ or 150 μM in Heart_Mit_. PTP closing was induced with 30 μM EGTA in Liver_Mit_ or 150 μM in Heart_Mit_ at 30, 60, 90, 180, and 240 s after Ca^2+^ was added. Data are mean ± SD *n* = 3, no statistical differences were observed.

In addition to releasing ions from the matrix, PTP has been proposed to work as a physiological uncoupling mechanism that prevents mitochondrial ROS over‐production (Morales‐García et al., 2021). Here, in Liver_Mit_ PTP opening did not affect ROS concentration, but instead, after 2–4 min ROS increased slightly as compared to the control (Figure 5A). In contrast, in Heart_Mit_ PTP opening slightly decreased ROS, although this was not statistically significant (Figure 5B). Further experiments are needed to determine whether in the heart PTP opening does decrease ROS production. A considerable decrease in ROS was expected (Kamunde et al., 2018). Further experiments subjecting mitochondria to different sources of stress have to be conducted to analyze this phenomenon.

## 4 DISCUSSION

Mitochondrial structure, density and function may vary from tissue to tissue (Veltri et al., 1990; Heine & Hood, 2020). Physiological activities such as ROS generation, membrane potential, and mitochondrial redox status may also vary with the tissue and the metabolic status (Kuznetsov et al., 2004). Mitochondrial responses to substrates, inhibitors or stress may be different (Kristián et al., 2002). Brain mitochondria contain fewer and smaller mitochondria that kidney or liver, while the heart has the largest and most abundant mitochondria (Veltri et al., 1990). In heart and liver the mitochondrial reticulum organization is different (Aon et al., 2006; Kang et al., 2024). Even at the level of individual enzymes, the tissue‐specific expression of subunit in Cytochrome c oxidase reflects different basal energy requirement in each organ (Vogt et al., 2021), and the Ca^2+^ efflux (Serna et al., 2022). In regard to the mitochondrial calcium uniporter MUC, expression of the regulatory subunits MICU1 and MICU2 is higher in the liver than in the heart, which results in slower Ca2+ uptake kinetics by the liver (Paillard et al., 2017).

In human heart myocytes, Ca^2+^ floods the cytoplasm 72 to 80 times per min and during intense cardiac activity heartbeats may increase to 160/min (Cheng & Lederer, 2008). Under these conditions, Ca^2+^ saturates the mitochondrial matrix, so in an effort to empty matrix Ca^2+^, PTP opens and depolarizes the membrane. Then, PTP needs to be rapidly closed in order to avoid ATP depletion, OMM‐disruption, Cytochrome *c* release and eventually, cell death (Kim et al., 2003; Morciano et al., 2021). In contrast, organs such as the liver are not subjected to these frequent depolarizations. Sometimes these tissues substitute Ca^2+^ uptake as second messenger using cAMP‐dependent signaling (Rodgers, 2022; Wahlang et al., 2018), so mitochondrial Ca^2+^ overload seems to be less likely, that is Liver_Mit_ face lower risk of Ca^2+^ flooding and thus PTP does not need to be as dynamic. To evaluate this idea, it was decided to compare the robustness of the mitochondrial‐PTP from liver versus that from heart.

Studies on whole tissues cells would complement our results (Agarwal et al., 2017; Boyman et al., 2019; Lu et al., 2016). In‐situ mitochondrial studies can detect organelle heterogeneity within the cell or in a given tissue, for example in the liver, periportal mitochondria behave different to those in peripheral hepatocytes (Azimzadeh et al., 2016). Low‐throughput analysis of small tissue/cell culture samples has proven useful to study mitochondrial heterogeneity and dysfunction within a tissue (Bury et al., 2020; Liao et al., 2020). In isolated mitochondria, it is not possible to collect information on activity modulation by surrounding organelles or by the differences of irrigation and hormone concentrations in the cytoplasm (Hoek et al., 1997; King et al., 2024). Still, isolated mitochondria are needed to elucidate specific catalytic mechanisms in mitochondrial proteins (Horten et al., 2024; Koch et al., 2024). In addition, some substrates and inhibitors do not traverse the plasma membrane readily and thus tight control of in‐situ mitochondrial activities is harder to achieve (Azimzadeh et al., 2016; Picard et al., 2011). There are advantages and drawbacks for studies on each in‐situ and ex‐vivo mitochondria. Most times, data are complementary.

When PTP behavior was compared in mitochondria isolated from either liver or heart, reversibility of PTP opening was lost earlier and with less Ca^2+^ in the liver, as 30 μM Ca^2+^ opened PTP, increasing the rate of oxygen consumption in state 4 (Figure 1A) and inducing mitochondrial swelling (Figures 2A and 3A). Heart_Mit_ PTP remained reversible for much longer time (Figures 1B, 2B, 3B and 4B).

Ca^2+^ retention and mitochondrial swelling assays showed that even though both mitochondria are capable of loading Ca^2+^ into the matrix (Belosludtsev et al., 2020; Coll et al., 1982; Crompton et al., 1987), the capacity of Heart_Mit_ is greater (Figure 4B) as compared to Liver_Mit_ (Figure 4A). This is in agreement with reports indicating that heart mitochondrial PTP withstands as much as 250 μM Ca^2+^ before opening (Azzolin et al., 2010; Halestrap, 2004; Korge et al., 2011) while liver mitochondrial PTP opens at 650 nM of Ca^2+^ (Belosludtsev et al., 2020; Chalmers & Nicholls, 2003).

Reversibility of the permeability transition (PT) is vital as the drop in ΔΨ leads to depletion of ATP and outer mitochondrial membrane disruption (Kim et al., 2003), which in turn leads to cell death. In Liver_Mit_, PT reversibility was lost sometime after 30 s (Figure 3A) or three sequential additions of 5 μM Ca^2+^ (Figure 4A). In contrast in Heart_Mit_ PT remained reversible for at least 2 min (Figure 3B) and withstood as much as eight consecutive 20 μM Ca^2+^ additions (Figure 4B,C). These experiments clearly show that PTP is much more resistant to Ca^2+^ in the heart than in liver mitochondria.

Under the conditions tested, ROS concentrations did not change significantly. In heart mitochondria small decreased was observed during the first 3 min, which returned to control at 4 min. ROS variation might be enhanced if mitochondria are subjected stress and then PT is evoked (Bernardi et al., 2023; Brady et al., 2004; Odagiri et al., 2009). However, this will remain as a perspective of this study.

Mitochondrial PTP structure and physiology may vary widely in different organisms. The *Debaryomyces hansenii* PTP is closed by monovalent cations (Cabrera‐Orefice et al., 2015). In *Yarrowia lipolytica*, it has been necessary to use ionophores and high Ca^2+^ loads to force PT (Kovaleva et al., 2009). Also, in crustaceans such as *Artemia franciscana*, *Litopenaeus vannamei*, *Lipidophtalmus louisianensis*, *Crangon crangon*, and *Palaemon serratus* it has not been possible to induce PT by Ca^2+^ overloading (Holman & Hand, 2009; Konrad et al., 2012; Menze et al., 2005; Rodriguez‐Armenta et al., 2021). In regard to structure, elimination of individual proteins has not led to conclusive results (Azzolin et al., 2010; Hurst et al., 2017; Izzo et al., 2016; Zoratti & Szabò, 1995). Recently it has been proposed that at least the F_1_F_o_‐ATPase and the adenine nucleotide transporter (ANT) form pores with differen sensitivities to inhibitors (Carrer et al., 2021). In fact both proteins or at least the ANT plus some subunits from the F_1_F_o_‐ATPase need to be present in order to constitute a PTP (He et al., 2017; Neginskaya et al., 2022, 2023). Our results show that two different tissues from the same organism display different PTP behavior. This would not be exceptional, for example different uncoupling proteins (UCPs) with different physiological roles are expressed in different tissues (Schulz & Schlüter, 2023; Wu et al., 2019; Zhang et al., 2010) so it seems worth exploring whether there are tissue‐specific differences in the structure/physiology of the mitochondrial ANT. In fact, in the heart, most ANT is isoform 1, while in the liver ANT isoform 2 is preponderant (Stepien et al., 1992). Even if these isoforms are quite similar in sequence, post‐transcriptional differences have been reported. Thus, the isoform‐specific properties of ANTs and their participation in mitochondrial PT would be worth exploring.

It is suggested that PTP function/regulation is tissue specific. In the heart, it may work as a physiological uncoupling system that detoxifies Ca^2+^ and decreases ROS production (Boyman et al., 2019; Lu et al., 2016). This has been described in the yeast *S*. *cerevisiae* (Cabrera‐Orefice et al., 2015; Guerrero‐Castillo et al., 2012; Morales‐García et al., 2021). The physiologic or structural basis for the difference in PTP behavior observed in each tissue is an interesting idea that complements data of widely different PTP regulation and function among species (Azzolin et al., 2010; Frigo et al., 2023). It would be interesting to explore if the frequent depolarization observed in cardiac myocytes favors association of proteins such as the Ca^2+^ uniporter, the ANT, the F_1_F_O_‐ATPase and possibly others while in other tissues in the same organism associations are different or non‐existent. In this regard, at least the Ca^2+^ antiport‐driven efflux is different in heart where the counter‐ion is Na^+^ than in the liver where it is H^+^ (Carafoli, 1987).

## AUTHOR CONTRIBUTIONS

Conceptualization, SUC, ARR. Experimental design SUC, ARR, CRG, Experimental work: CRG, ARR, OMR, EMM & MRB, Writing SUC, CRG. Editing ARR, SUC, CRG, MRB & OMR. Project funding, SUC.

## FUNDING INFORMATION

This research was partially funded by research grants to SUC: CONAHCYT CF2023‐I‐199; DGAPA/PAPIIT UNAM IN208821 and DGAPA/PAPIIT UNAM IN211224; OMR has a Postdoctoral fellowship from CONAHCYT CVU 639365; EMM is a MsC CONAHCYT fellow CVU 1184243 enrolled in the Ciencias Bioquímicas Program at UNAM; NAM; CRG is a PhD CONAHCYT fellow, CVU 966402 enrolled in the Ciencias Bioquímicas Program at UNAM; MRB is an undergraduate CONAHCYT fellow CVU 1202206.

## CONFLICT OF INTEREST STATEMENT

The authors declare that they have no known competing financial interests or personal relationships that could have appeared to influence the work reported in this paper.

## ETHICS STATEMENT

The use of animals at the Instituto Nacional de Cardiología was authorized by the Committee for the Care and Use of Laboratory Animals (C.I.C.U.A.L.) with protocol number INC/CICUAL/008/2020. At the Instituto de Fisiología Celular (UNAM), a local committee approved all experimental procedures according to Ethics in Animal Experimentation (SUV124‐19).

## Supporting information


Figure S1.

## Data Availability

All experiments were conducted at least in triplicates and the raw data are available from us on request.
