# Supplementary material for: Tissue‐specific differences in Ca2+ sensitivity of the mitochondrial permeability transition pore (PTP). Experiments in male rat liver and heart
Source: Physiol Rep. 2024 May 22;12(10):e16056. doi: 10.14814/phy2.16056 (PMC11111423; doi:10.14814/phy2.16056)
Supplement: Supplementary file 1 — Figure S1. [file PHY2-12-e16056-s001.docx]

Supplementary Fig. S1.


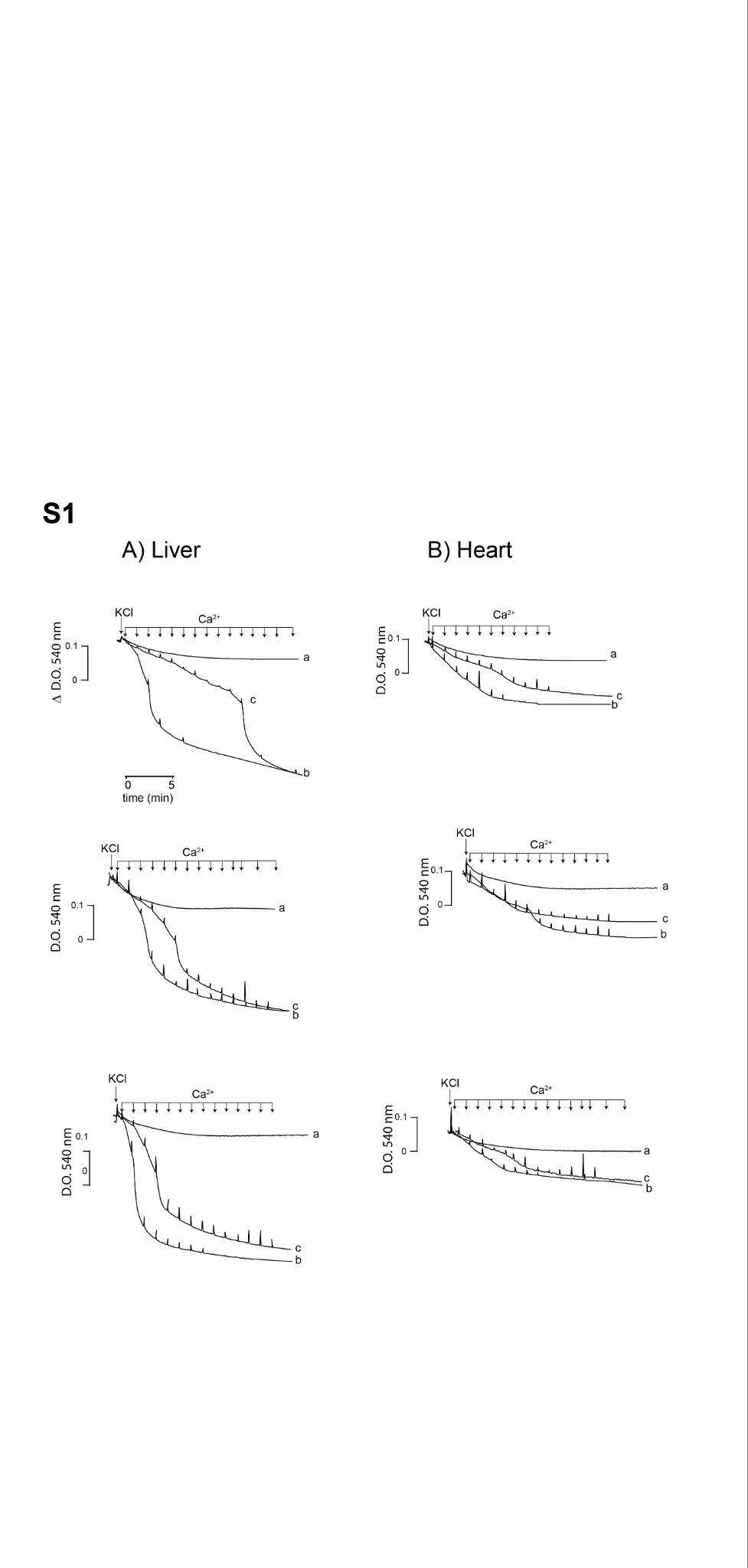


Supplementary Fig. Si Mitochondrial (M) PT swelling in (A) Liver_mit_ and (N) Heart_mit_. Raw data from three experiments. Reaction mixture as in Fig. 1.


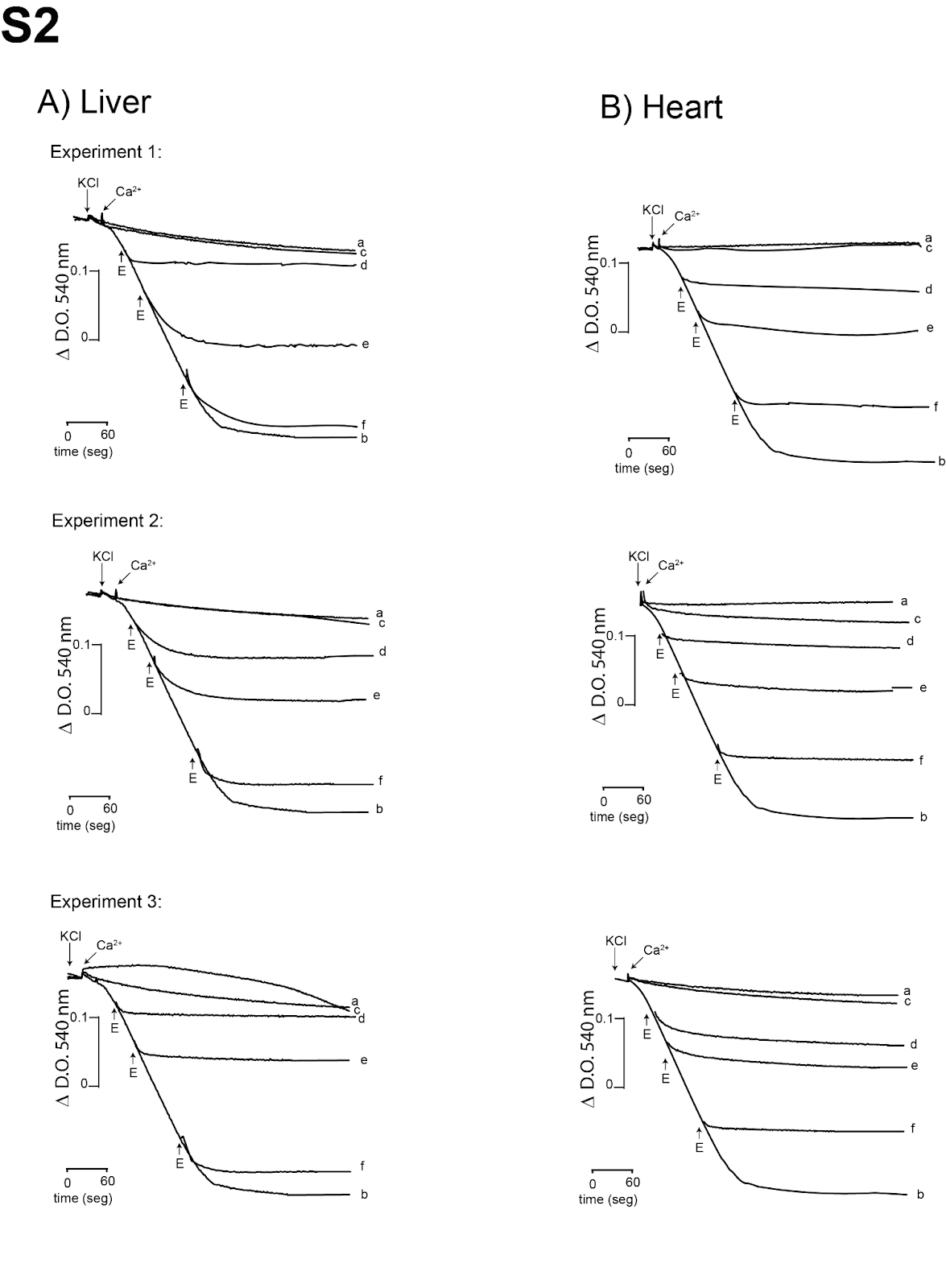


Supplementary Fig. S2. Mitochondrial (M) PT reversibility in (A) Liver_Mit_ and (B) Heart_Mit_ Raw data from three experiments. Reaction mixture as in Fig. 2.


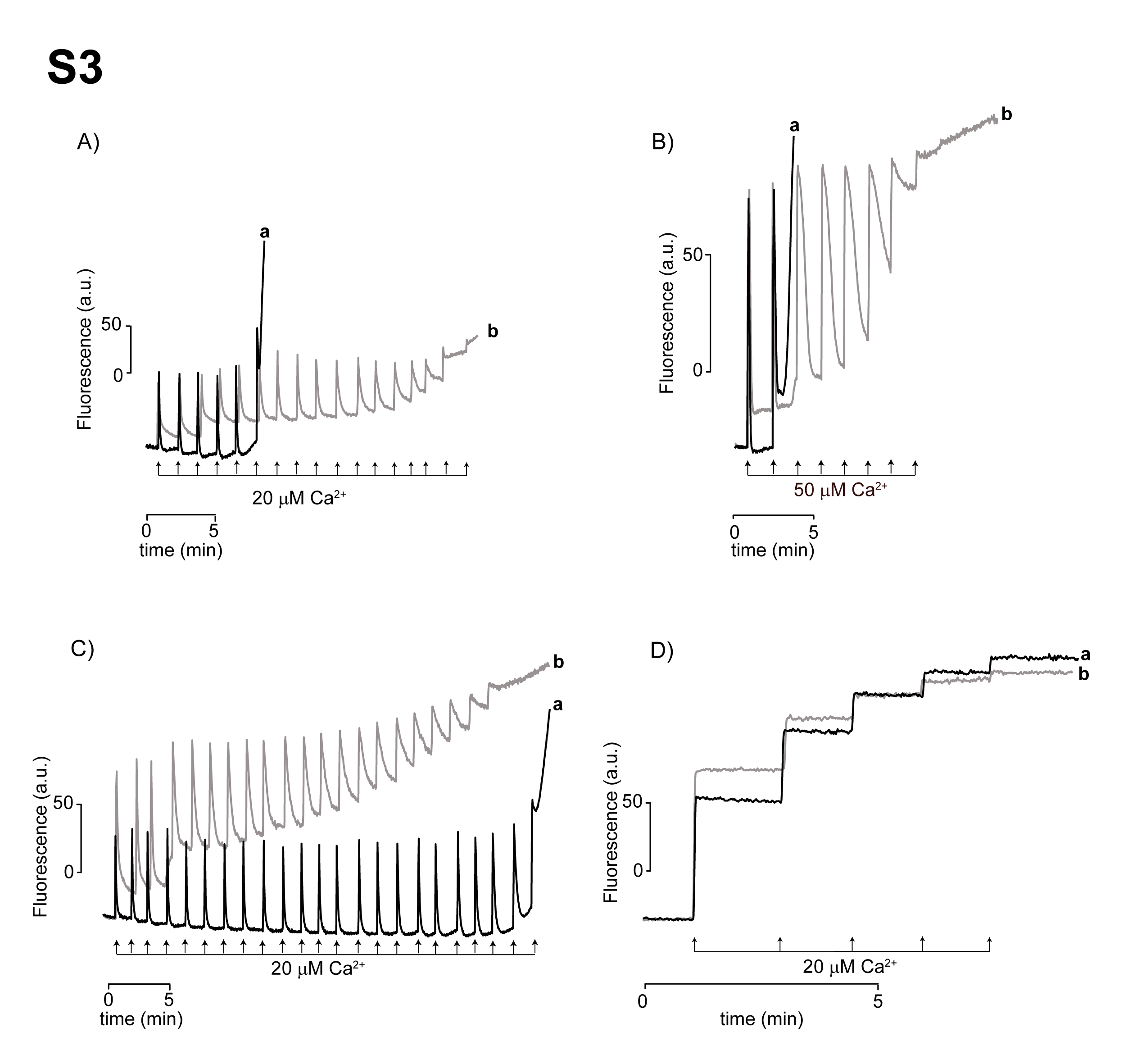


Supplementary Figure S3. Effect of CsA and RuR on Ca^2+^ uptake kinetics in mitochondria from either liver (traces a) or heart (traces b) as measured by Ca^2+^ Green-5N fluorescence. Reaction mixture, 150 mM sucrose, 50 mM KCl, 5 mM Tris, and 250 μM KH_2_PO_4_ pH 7.4. As substrate 10 mM/10 mM glutamate/malate. Fluorescent probe 100 nM Calcium Green-5N. As indicated, for each figure, sequential Ca^2+^ additions were 20 μM (A, C & D) or 50 μM (B) CaCl_2_ at 1.5 min intervals until PT induction. C In the presence of 5 μM CsA, an upward drift was observed in Heart_Mit_, probably due to the presence of KCl. and D: 74 μM RuR and the Y axis is [Ca^2+^]. In all traces a (black line) is for Liver_Mit_ and b (gray line) for Heart_Mit_.
